# High Quality Genome-Wide Genotyping from Archived Dried Blood Spots without DNA Amplification

**DOI:** 10.1371/journal.pone.0064710

**Published:** 2013-05-30

**Authors:** Krystal R. St. Julien, Laura L. Jelliffe-Pawlowski, Gary M. Shaw, David K. Stevenson, Hugh M. O’Brodovich, Mark A. Krasnow, the Stanford BPD Study Group

**Affiliations:** 1 Department of Biochemistry and Howard Hughes Medical Institute, Stanford University School of Medicine, Stanford, California, United States of America; 2 Department of Pediatrics, Stanford University School of Medicine, Stanford, California, United States of America; 3 California Genetic Disease Screening Program of the California Department of Public Health, Richmond, California, United States of America; 4 Department of Epidemiology and Biostatistics, University of California San Francisco, San Francisco, California, United States of America; Yale School of Public Health, United States of America

## Abstract

Spots of blood are routinely collected from newborn babies onto filter paper called Guthrie cards and used to screen for metabolic and genetic disorders. The archived dried blood spots are an important and precious resource for genomic research. Whole genome amplification of dried blood spot DNA has been used to provide DNA for genome-wide SNP genotyping. Here we describe a 96 well format procedure to extract DNA from a portion of a dried blood spot that provides sufficient unamplified genomic DNA for genome-wide single nucleotide polymorphism (SNP) genotyping. We show that SNP genotyping of the unamplified DNA is more robust than genotyping amplified dried blood spot DNA, is comparable in cost, and can be done with thousands of samples. This procedure can be used for genome-wide association studies and other large-scale genomic analyses that require robust, high-accuracy genotyping of dried blood spot DNA.

## Introduction

Newborn screening programs around the world use Guthrie filter cards to preserve several spots of blood obtained from heel pricks of newborn infants. These samples are used to screen infants for metabolic and genetic diseases such as phenylketonuria and cystic fibrosis, and they are stored as dried blood spots (DBS). Some states have archived these specimens for many years, and California currently accrues DBS from over a half million newborns each year.

Although DBS can be archived for decades, each DBS is a limited resource containing only ∼50 µl of blood. The original protocol for newborn screening of phenylketonuria utilized a portion of a DBS for the test [1], and although DBS are currently used to screen for many diseases, technical advances permit all of these tests to be conducted on a similar portion of a DBS. After McCabe et. al [2] reported that genomic DNA (gDNA) could be extracted from DBS, scientists began to explore methods to amplify and analyze the minute quantities of gDNA. Many PCR-based amplification analyses have shown that DNA fragments of hundreds to thousands of base pairs can be amplified from gDNA extracted from a small portion of a DBS [3], [4], [5] and probed for single nucleotide polymorphisms (SNPs) or gene deletions [6], [7]. More recently, it was shown that targeted SNP analysis can be done on whole genome amplified DNA (wgaDNA) from DBS [8], [9], [10], [11], [12] and that wgaDNA from DBS can be used for array-based genome-wide SNP genotyping [13], [14], [15], [16].

Here we show that genome-wide SNP genotyping can be performed using small amounts of unamplified gDNA extracted from a portion of a DBS. We find that genotyping with gDNA is more robust than genotyping with corresponding wgaDNA, and show that our protocol can be used to accurately genotype thousands of samples of archived DBS. We discuss the benefits of genotyping gDNA and suggest when gDNA from DBS should be used instead of wgaDNA for genetic analyses of DBS.

## Methods

### Ethics Statement

This study was approved by the Committee for the Protection of Human Subjects of the Health and Human Services Agency of the State of California and the Institutional Review Board of Stanford University.

### Study Population and Samples

This study was carried out to determine if genomic DNA extracted from DBS could be used for genome-wide SNP genotyping in a case-control study investigating genetic associations with bronchopulmonary dysplasia (BPD), a chronic lung disease of preterm infants. Infants with BPD (cases) and infants without BPD (controls) were identified from the California Perinatal Quality Care Collaborative database (CPQCC; www.cpqcc.org) [17]. The Genetic Disease Screening Program (GDSP) within the California Department of Public Health (CDPH) linked each subject’s CPQCC information to their archived DBS, which was collected as part of the state newborn screening program. Using identifiers present in both datasets and in birth certificate data maintained by CDPH, 96.0% of BPD cases (n = 1047) and 90.3% of controls (n = 960) identified by CPQCC were linked to newborn screening records. The personal health information variables used for linkage were not provided to the investigators. Of the linked samples, DBS of 91.3% of cases (n = 996) and 87.2% of controls (n = 927) were obtained from storage. Among the obtained samples, 74 cases (6.8%) and 74 controls (7.0%) could not be used because of CDPH guidelines (e.g., only a single DBS remained in storage). Thus, DBS from 1,775 cases and controls were received and available for extraction, and gDNA was isolated from 1,773 (921 cases and 852 controls).

### DNA Extraction, Amplification, and Quantification

A detailed description of the extraction protocol is provided in Text S1. Each DBS is approximately 1.2 cm in diameter and provides ∼30 separate 2 mm diameter punches, which we obtained using a 2 mm mouse ear punch (Kent Scientific). DNA extractions performed during the protocol development phase were done using two to five separate 2 mm diameter punches per DBS. For the Genome-wide Association Study (GWAS), five punches per DBS were used in an initial extraction, and additional punches from the same DBS were used as necessary in subsequent extractions to reach a minimum of 170 ng gDNA (17 µl of 10 ng/µl). These additional punches and extractions are referred to as “iterations”.

For whole genome amplification, the multiple displacement method of DNA amplification was performed using the Genomiphi V2 DNA amplification kit (GE Healthcare) on 1 µl (2.7–22 ng) of starting gDNA.

We quantified DNA using the Qubit High Sensitivity (HS) Assay kit (Invitrogen), which has a range of 0.2 ng to 100 ng. All measurements were done using 1 µl of DNA added to 199 µl of assay buffer prepared as directed by the manufacturer. The 200 µl sample was transferred to a Qubit Assay Tube and measured with the Qubit 1.0 Fluorometer. DNA concentrations were also determined by Illumina on 1–2 µl of each DNA sample using the Quant-iT Picogreen dsDNA Assay (Invitrogen), which has an assay range of 50 pg to 2 µg. Although the DNA concentrations determined by Illumina using the Picogreen assay correlated with the concentrations we determined by the Qubit assay, they were generally smaller by ∼30% (unpublished data).

### Genotyping

All DNA samples isolated from DBS were genotyped by Illumina (San Diego, CA) as described below. Illumina requested a minimum of 20 µl of 50 ng/µl (1 µg total) of DNA for genotyping. Each DNA sample we provided was resuspended in 20 µl of 10 mM Tris-HCl (pH 8)/1 mM EDTA (TE buffer), and then 1 µl of sample was removed for DNA quantification by the Qubit assay. When iteration steps were used, ∼1 µl of sample was typically lost due to incomplete pipetting or evaporation. Another 1 µl of sample was removed for a second round of DNA quantification by the Qubit assay. We therefore provided ∼17 µl of DNA per sample to Illumina. Illumina then used 1–2 µl per sample for Picogreen DNA quantification and 3–4 µl per sample for genotyping. The 17 µl provided ensured that Illumina could perform up to two Picogreen DNA quantification analyses and three genotyping runs in case of DNA quantification or genotyping failures.

Genome-wide SNP genotyping of the provided DNA samples was done by Illumina using their Infinium single nucleotide extension SNP genotyping assay [18] and a cytoSNP or Omni bead microarray [19], [20]. For quality control, at least two Illumina control DNA samples were genotyped in parallel with every 94 experimental DNA samples we provided. Genotyping during the protocol development phase used a proprietary Illumina cytoSNP bead microarray designed to analyze approximately 300,000 loci. Genotyping during the GWAS discovery phase and follow-up analysis of 81 poorer-performing samples used the HumanOmni2.5 BeadChip designed to analyze 2,443,177 loci. The microarrays were scanned with Illumina iScan, and the obtained values were analyzed with the Genotyping Module of GenomeStudio software (Illumina) [21]. GenomeStudio provides raw data normalization, clustering, and genotype calling, and calculates a GenCall score for each genotype, a measure of the accuracy of the genotype call [22]. For all analyses, the minimum GenCall score allowed was set to 0.1. Because of the small number of samples analyzed in the protocol development phase, GenomeStudio analysis of an Illumina training set was performed first and clustering from the training set was used in the analysis and genotype calling of our test samples. Genotype calling of the GWAS discovery dataset was based on clustering performed with the 1,773 GWAS DNA samples themselves; no re-clustering was done for the follow up analysis of the 81 poorer performing samples.

### Evaluation of Genotyping Performance

Genotyping performance of each sample was assessed by call rate (CR), the ratio of SNP calls made for a sample to total possible SNP calls, and 10% GenCall score (10GC), the tenth percentile of GenCall scores across all successfully genotyped loci in the sample [19], [22], [23]. CR and 10GC were determined using GenomeStudio software. In addition, we assessed the following parameters: *SNP genotyping failure rate*, the ratio of failed SNP calls to total possible SNP calls; *SNP genotyping replication rate*, the ratio of consistent SNP calls in the original and duplicate samples to the total number of SNPs called in both samples; and *SNP genotyping replication error*, the ratio of discrepant SNP calls to the total number of SNPs called in both samples. The latter was used to estimate genotyping error.

### Confidentiality Restrictions on Sharing Genotype Data

In adherence with confidentiality requirements by the California Department of Public Health regarding genotype data derived from DBS used for this study, individual genotypes cannot be shared beyond the investigative team or the scope of the approved study. Hence, ascertained genotypes have not been deposited in public databases and no plans are in place or envisioned to facilitate such sharing beyond contacting the primary investigator of the Stanford BPD Study Group (Dr. O'Brodovich) directly.

## Results

### A High Throughput Method for Genomic DNA Isolation from Dried Blood Spots

We modified an established proteinase K digestion and isopropanol DNA precipitation protocol for extraction of genomic DNA (gDNA) from DBS [24]. To increase throughput, we made two significant modifications to the protocol. First, we switched from a single tube to a 96 well format, which allowed simultaneous processing of up to four 96 well plates. This provided a 16-fold increase in throughput. Second, we shortened the overnight incubations with proteinase K and isopropanol to one hour incubations, increasing throughput another 3-fold. Together these modifications provided an overall 48-fold increase in throughput.

### Robust Genome-wide SNP Genotyping can be Done with Unamplified Genomic DNA from Dried Blood Spots

**Figure 1:**
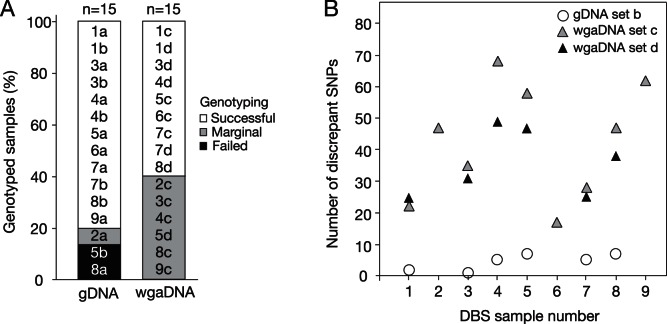
Success and accuracy of genotyping with genomic or amplified DNA from dried blood spots. Genomic DNA (gDNA) extracted from two 2 mm punches obtained from archived dried blood spots (DBS) from nine individuals (1–9) was genotyped on an Illumina Infinium 300,000 SNP test chip (set “a”, 1a–9a). For six of the DBS (1,3,4,5,7,8), a set (set “b”) of extraction duplicates (duplicate extractions from the same DBS) was prepared and genotyped in the same way. A 1 µl aliquot of each of these 15 samples of gDNA was whole genome amplified (wgaDNA) and then genotyped in the same way as sets “a” and “b”: set “c” wgaDNA samples (1c–9c) were amplified from set “a” gDNA samples, and set “d” wgaDNA samples (1d, 3d, 4d, 5d, 7d, 8d) were amplified from set “b” gDNA samples. (A) Performance quality of the gDNA and wgaDNA samples on genome-wide SNP genotyping. Successful, Marginal, and Failed sample performance parameters are given in Results. (B) SNP genotyping replication error. The number of SNP discrepancies for each sample was determined using gDNA set “a” as the reference. Replication error (the number of discrepancies among SNPs called in both the sample and reference divided by the total number of SNPs called in both) was 1.6×10^−5^ for set “b”, 1.5×10^−4^ for set “c”, and 1.2×10^−4^ for set “d”. These replication error values are provisional because we do not know the true genotype of the discrepant loci and there may be gDNA-specific and/or wgaDNA-specific artifacts.

During the protocol development phase, we extracted gDNA from two 2 mm punches of a DBS from nine individuals to test the quality of isolated, unamplified gDNA. We also used a separate set of punches to extract a second set of gDNA from six of the nine DBS to generate gDNA extraction duplicates. Each sample was genotyped using the Illumina Infinium platform [18] and a proprietary 300,000 locus bead microarray. Sample performance was assessed based on call rate (CR) and 10% GenCall score (10GC) determined by GenomeStudio software [19], [21], [22], [23]. Of the 15 gDNA samples genotyped, 12 were considered successful (CR >99%, 10GC >0.7), one was marginal (CR >99%, 10GC <0.7), and two failed (CR <99%; Figure 1A). For each of the 15 gDNA samples, we amplified 1 µl of DNA (2.7–22 ng) to produce whole genome amplified DNA (wgaDNA) controls that we genotyped in parallel with the corresponding gDNA samples. Of the 15 wgaDNA samples genotyped, nine were successful, six were marginal, and none failed (Figure 1A). There were more failed SNP genotyping calls with the wgaDNA samples (10,125±622 (mean ± S.D.) failed calls out of 299,140 genotyped loci; SNP genotyping failure rate = 0.034) than with the gDNA samples (8,248±636 failed calls; SNP genotyping failure rate = 0.028; p = 2.6×10^−7^, Student’s t-test). There was also a 9-fold greater SNP genotyping replication error for the 15 wgaDNA duplicates (1.4×10^−4^) than for the six gDNA duplicates (1.6×10^−5^) when compared against the SNP calls of the original nine gDNA samples (Figure 1B, Table 1).

**Table 1:** Comparison of genomic DNA and whole genome amplified DNA from DBS.

		Portion of DBS	DBS per genotype run	Samples expected to reach 10 ng/µl	Time^1^	Cost per sample	SNP error rate^2^	SNP failure rate^3^	Useful for CNV analysis	Useful for DNA sequencing
gDNA	Initial extraction	17%	4%	37%	22 h	$1	1.6×10^−5^	0.028	Yes	Yes
	+1 iteration	33%	8%	72%						
	+2 iterations	50%	13%	93%						
	+3 iterations	67%	17%	99%	22 h	$1				
wgaDNA	DNA extraction and amplification	7%	2%	100%	24 h	$5	1.4×10^−4^	0.034	No	?

1 Time for two 96 well extraction plates, not including time to catalog samples; All iterations of samples from the original two 96 well plates typically fill two new 96 well plates (22 hours includes all iterations).

2 Mean SNP genotyping replication error rate from Figure 1B.

3 Mean SNP genotyping failure rate (protocol development sample set); gDNA and wgaDNA failure rates are significantly different (p = 2.6×10^−7^, Student’s t-test).

**Figure 2:**
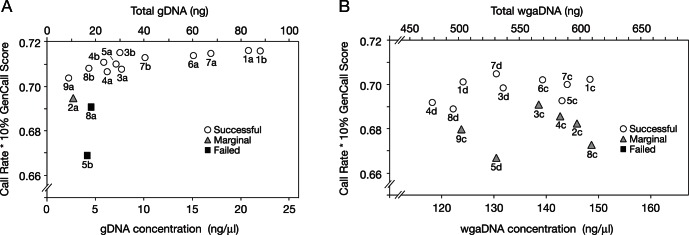
Genotyping performance as a function of gDNA concentration. The genotyping performance of the gDNA samples (A) and wgaDNA samples (B) described in Figure 1 is plotted against the DNA concentration of the sample as determined by Qubit for gDNA and Picogreen for wgaDNA (bottom axis) and total amount of DNA used (4 µl) during each genotyping run (top axis). Picogreen was used for wgaDNA because Qubit does not discern concentrations above 100 ng/µl. Note that all gDNA samples with DNA concentrations above 5 ng/µl (∼20 ng DNA) were genotyped successfully (A). No similar threshold was observed for wgaDNA samples (B).

All gDNA samples with a concentration above 5 ng/µl (∼20 ng DNA per genotyping run) were successfully genotyped (Figure 2A). No relationship between sample performance and DNA concentration was observed for the wgaDNA samples in the DNA concentration range tested (Figure 2B).

### Different Dried Blood Spots Yield Different Amounts of DNA

**Figure 3:**
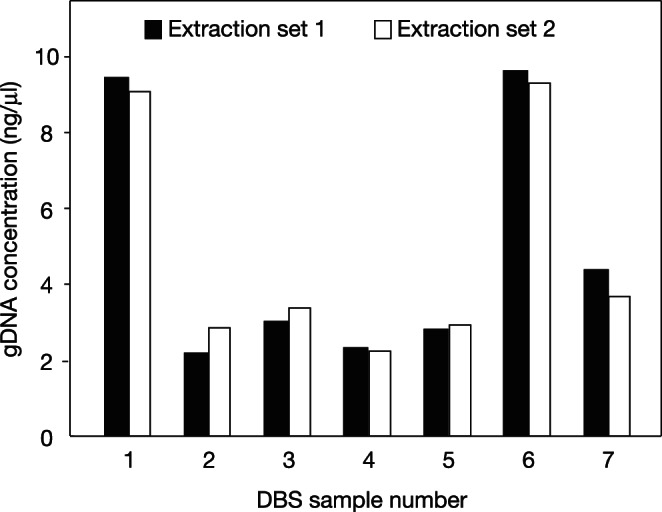
Reproducibility of gDNA yields from the same dried blood spot. gDNA was extracted from two 2 mm punches of seven different archived DBS (1–7), and the process was repeated with fresh punches from the same DBS on a separate day. DNA was quantitated by Qubit. Different DBS showed ∼5-fold difference in DNA yield, whereas extraction duplicates of the same DBS gave almost identical DNA yield (103% ±15%, mean ± S.D.).

A test of seven gDNA DBS extractions and seven corresponding extraction duplicates showed that gDNA yield differed by nearly 5-fold (approximately 2–10 ng/µl) across different DBS (Figure 3). Extraction duplicates of the same DBS, however, gave nearly equivalent yields (Figure 3), demonstrating that differences in gDNA yield were due to differences among the DBS and not variability in the extraction procedure. We observed an even wider range (0 ng to 62 ng) of gDNA yields from a single extraction (before iterations) across different DBS in our Genome-Wide Association Study (GWAS) discovery samples (Figure S1A, C). There was no relationship between gDNA yield and the gestational age or birth weight of the individual from which the DBS was obtained (Figure S1).

### Extracting Larger Portions of Low Yield Dried Blood Spots to Obtain Sufficient gDNA for Genotyping

**Figure 4:**
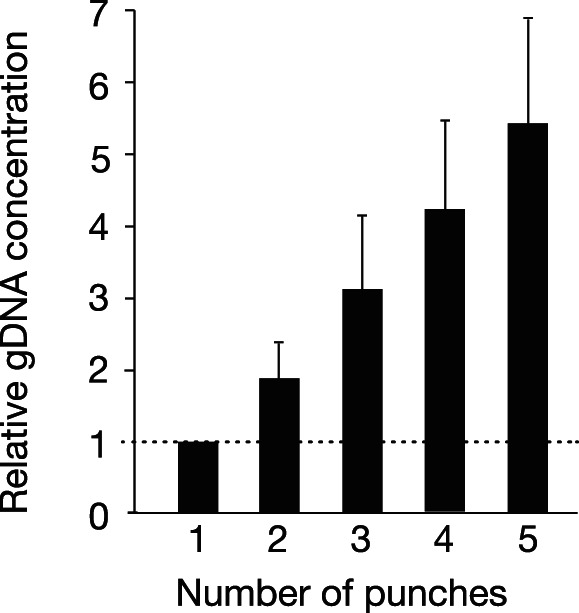
gDNA yield increases with increasing number of dried blood spot punches. The indicated number of punches was obtained from 19 archived DBS for gDNA extraction. DNA yield was determined by Qubit (Table S1) and the values (mean ± S.D.) were normalized to the yield for a single punch. p<0.0001, one-way ANOVA.

We extracted gDNA from 19 different DBS using one to five 2 mm punches from each DBS. The overall DNA yield increased linearly with the number of punches used (Figure 4), though again there was a wide range (7-fold) in the amount of gDNA obtained per sample (Table S1). Although samples containing as little as 20 ng gDNA (4 µl of 5 ng/µl) were successfully genotyped (Figure 2), a target minimum of twice that concentration (10 ng/µl gDNA for each sample, corresponding to genotyping runs using ∼40 ng gDNA) was used to be conservative. In our final protocol, we used five 2 mm punches from each DBS (∼17% of a DBS) for gDNA extraction and achieved the target concentration of ≥10 ng/µl of gDNA for 37% (n = 648) of the samples in a single extraction. For the samples that failed to achieve the target concentration in the first extraction, we calculated the number of additional sets of five 2 mm punches from each DBS that would be needed to reach the target and then carried out extractions (iterations) with the additional sets. This required up to three additional sets (15 additional punches), which accounts for ∼50% of the DBS (Table 1). The iterations were carried out in the same way as the initial extraction, except that the original extraction sample was used to resuspend the DNA pellet(s) rather than fresh TE buffer in order to avoid increasing final sample volume. With this protocol, 86% of samples achieved the target DNA concentration. Due to the variability in DNA yield with multiple extractions of the same DBS (Figure 3), some samples did not reach the 10 ng/µl target. Despite this, nearly all of the samples (99.3%) that did not reach 10 ng/µl were genotyped successfully.

### Genomic DNA from Dried Blood Spots can be used for GWAS

The 1,773 GWAS samples (921 cases and 852 controls) containing ∼17 µl gDNA (0 ng/µl to 67.2 ng/µl) were genotyped at 2,443,177 loci with the HumanOmni2.5 BeadChip on the Illumina Infinium platform. Among the 1,773 samples genotyped, 1,712 samples (96.6%) were considered successful (CR>99%, 10GC>0.601), 22 (1.2%) were marginal (CR>99%, 10GC<0.601), and 39 (2.2%) failed (CR<99%). Of the 1,712 successful samples, 1,688 (98.6%) met the more conservative CR threshold of ≥99.5%. Of the 39 failed samples, 17 (44%) failed due to DNA contamination. Of the 2,443,177 loci on the HumanOmni2.5 BeadChip, 32,292 loci (1.3%) failed for all successful and marginal samples and were excluded from the above CR and 10GC calculations. The overall CR among the successful and marginal samples across the remaining 2,410,885 genotyped loci was 99.97%.

Based on analyses completed during the protocol development phase (Figure 2), we expected that the poorer performing samples would be those with the lowest gDNA concentrations, especially those that did not reach the 10 ng/µl target. However, neither DNA concentration nor other examined variables such as the subject’s birth weight, gestational age, disease (bronchopulmonary dysplasia) phenotype, or sex correlated significantly with genotyping performance (Table S2). Repeat gDNA extraction and genotyping of 81 poorer performing samples (30 successful at the lower CR of 99–99.5%, 21 marginal, 17 failed, and 13 contaminated) showed that 69 (85%) had improved genotyping performance (Table S3). Forty-three of the 51 (84%) samples that were marginal, failed, or contaminated in the original genotyping were successful on repeat extraction and genotyping (Table S3). We conclude that nearly all of the archived DBS can be genotyped successfully with this protocol.

Analysis of five extraction duplicates (duplicate extractions from the same DBS) and five genotyping duplicates (duplicates produced from one extraction preparation) showed a replication rate of over 99.99% for each pair (Table S4). The number of discrepant SNPs across extraction duplicates was not significantly different from the number of discrepant SNPs across genotyping duplicates (p = 0.55, Student’s t-test), showing that the extraction protocol does not introduce additional error.

### Time and Cost Estimates for Genomic DNA Extraction

To carry out the gDNA extractions for the GWAS study, it took one individual half of one work week (22 hours) to obtain punches and isolate gDNA from 192 DBS using the protocol detailed in Text S1. Reagents cost ∼$2 per DBS (Table 1 and Table S5). For each plate of 96 extracted DBS, approximately one additional plate of iteration extractions was needed for the 63% of samples that did not meet the 10 ng/µl target. Personnel costs (∼$10 per DBS) and reagent costs (∼$2 per DBS) for the gDNA extraction steps are small relative to the cost of genome-wide SNP genotyping, which cost ∼30 times more than personnel and reagent costs combined.

## Discussion

We have developed a 96 well protocol for gDNA extraction that provides sufficient DNA for robust, high accuracy, genome-wide SNP genotyping using a portion (17–67%) of an archived DBS. The 96 well format and other modifications yielded a 48–fold increased throughput relative to the published gDNA extraction protocol on which our protocol was based [24]. The increased throughput allowed one individual to isolate gDNA from over 1,700 archived DBS in less than 2 months for use in genome-wide SNP genotyping for a GWAS (Wang et al., submitted). Nearly all of the gDNA samples (>96.5%) were successfully genotyped on Illumina’s HumanOmni2.5 BeadChip, and analysis of extraction and genotyping duplicates gave replication frequencies of >99.99%.

There was a ∼60-fold range in gDNA yield from archived DBS from different individuals. This was not due to variability in the extraction procedure as duplicate extractions from the same DBS gave nearly identical yields (Figure 3). The wide range of gDNA yields presumably reflects differences in the number of nucleated cells (leukocytes) present in the blood of newborns from which the DBS were prepared [25], although differences in Guthrie card construction and gDNA stability during storage could potentially contribute [5], [26].

The wide range in gDNA yield from DBS is significant because the amount of gDNA used for genotyping is a strong predictor of performance on the Illumina Infinium platform: Figure 2 shows that successful genotyping performance was obtained with ∼20 ng (4 µl of 5 ng/µl gDNA) or more of unamplified gDNA. We chose 10 ng/µl as our target concentration to be conservative, and providing 17 µl to Illumina ensured that they would have extra sample for additional DNA quantifications or genotyping runs in case of pipetting or machine failures. Our target DNA yield is less than one fifth of the amount of DNA that Illumina regularly requests for Infinium SNP genotyping (1 µg DNA, provided as 20 µl of 50 ng/µl DNA, per sample to be genotyped). We obtained 170 ng or more of gDNA from 37% of all archived DBS using ∼17% (five 2 mm punches) of each DBS. Further DNA extractions (iterations) using up to 50% more of a DBS (15 2 mm punches) were required to reach the target amount for the remaining DBS.

Table 1 compares our procedure for gDNA isolation with wgaDNA isolation for genome-wide SNP genotyping of DBS. We initially thought DNA amplification would be essential because of the limited starting material available in archived DBS. Preparing wgaDNA does indeed allow one to use just a small fraction of a DBS (7%) for genotyping, about a third to a tenth less than that needed for gDNA. Preparing wgaDNA is also about twice as fast as preparing gDNA. Costs for the two methods are comparable because preparing wgaDNA has greater reagent costs but saves on labor (Table 1 and Table S5). For any application in which only a small fraction of a DBS is available, or DNA isolation time is critical, preparation of wgaDNA is preferred.

The main advantages of using unamplified gDNA are that genotyping is more robust (fewer failed SNP genotyping calls and lower genotyping replication error), and gDNA can be used for analyses that may be problematic with wgaDNA due to potential DNA amplification bias across regions of the genome. For example, we were able to perform copy number variation analysis on the gDNA samples from our GWAS and to carry out whole exome sequencing on gDNA isolated from a second set of archived DBS (unpublished data). For analyses that require high accuracy genotyping or sequencing, gDNA should be used. With the increasing sensitivities of next generation sequencing and other DNA analytical methods, many applications will likely require an even smaller portion of each DBS than the applications described here. The procedure described should enable large-scale, high accuracy genomic studies of archived DBS, while helping to preserve these precious resources.

## Supporting Information

Figure S1 gDNA yield from dried blood spots as a function of gestational age and birth weight. Five 2 mm punches were obtained from 1,773 archived DBS, gDNA was extracted, and DNA concentrations were determined by Qubit (A, C). Extraction iterations were completed as necessary and DNA concentrations of the final preparations were determined by Qubit (B, D). (A, B) Gestational age. (C, D) Birth weight.
(EPS)

Table S1 Effect on gDNA yield by the number of DBS punches used in the extraction.
(DOCX)

Table S2 Genotyping performance by sample DNA concentration and subject parameters.
(DOCX)

Table S3 Performance of 81 poorer performing gDNA samples on repeat DBS extraction and genotyping.
(DOCX)

Table S4 Replication rate in DBS extraction and genotyping duplicates.
(DOCX)

Table S5 Reagent costs for preparing gDNA and wgaDNA from DBS.
(DOCX)

Text S1 Dried Blood Spot (DBS) Genomic DNA Extraction Protocol.
(DOCX)
