# Supplementary material for: High Quality Genome-Wide Genotyping from Archived Dried Blood Spots without DNA Amplification
Source: PLoS One. 2013 May 30;8(5):e64710. doi: 10.1371/journal.pone.0064710 (PMC3667813; doi:10.1371/journal.pone.0064710)
Supplement: Table S1 — Effect on gDNA yield by the number of DBS punches used in the extraction. (DOCX) [file pone.0064710.s002.docx]

| **Table S1.** Effect on gDNA yield by the number of DBS punches used in the extraction | | | | | |
| --- | --- | --- | --- | --- | --- |
|  | gDNA yield (ng/μl) | | | | |
| DBS sample | 1 punch | 2 punches | 3 punches | 4 punches | 5 punches |
| 1 | 2.6 | 4.1 | 8.0 | 9.0 | **14*** |
| 2 | 0.65 | 1.3 | 2.7 | 4.4 | 5.2 |
| 3 | 1.7 | 4.1 | 8.4 | 7.6 | **12** |
| 4 | 1.2 | 1.6 | 5.1 | 4.9 | 5.3 |
| 5 | 2.9 | 5.4 | 3.5 | 8.6 | **14** |
| 6 | 2.7 | 5.3 | 7.6 | **14** | **14** |
| 7 | 1.6 | 3.9 | 5.2 | 5.6 | **12** |
| 8 | 0.49 | 0.88 | 0.96 | 2.0 | 3.0 |
| 9 | 0.99 | 1.8 | 2.5 | 2.8 | 3.8 |
| 10 | 3.5 | 5.1 | **12** | **15** | **15** |
| 11 | 8.1 | **13** | **29** | **24** | **23** |
| 12 | 1.5 | 4.8 | 5.5 | 8.7 | **12** |
| 13 | 1.3 | 2.1 | 3.1 | 4.2 | 6.9 |
| 14 | 3.3 | 4.4 | 8.5 | **17** | **19** |
| 15 | 2.7 | 5.0 | **13** | **15** | **12** |
| 16 | 2.0 | 1.8 | 3.4 | 5.4 | 9.3 |
| 17 | 2.0 | 3.4 | 5.0 | 6.7 | 7.8 |
| 18 | 2.2 | 5.4 | 7.4 | **13** | **15** |
| 19 | 0.74 | 1.3 | 2.0 | 3.0 | 3.0 |
| Samples >10 ng/μl | 0 | 1 | 3 | 6 | 11 |

* Bold, samples with >10 ng/μl gDNA
