# Supplementary material for: High Quality Genome-Wide Genotyping from Archived Dried Blood Spots without DNA Amplification
Source: PLoS One. 2013 May 30;8(5):e64710. doi: 10.1371/journal.pone.0064710 (PMC3667813; doi:10.1371/journal.pone.0064710)
Supplement: Table S2 — Genotyping performance by sample DNA concentration and subject parameters. (DOCX) [file pone.0064710.s003.docx]

| **Table S2**. Genotyping performance by sample DNA concentration and subject parameters | | | | | | | | | | | | | | |
| --- | --- | --- | --- | --- | --- | --- | --- | --- | --- | --- | --- | --- | --- | --- |
|  |  | DNA concentration (ng/μl)^2^ | | | Birth weight (g) | | Gest. Age^1^ (d) | | BPD^1^ | | | Gender | | |
| Genotyping  performance | DBS^1^  samples | Range | Mean^3^ | P^4^ | Mean^3^ | P^4^ | Mean^3^ | P^4^ | Case | Control | P^4^ | Female | Male | P^4^ |
| Successful | 1710 | 2.5 - 51 | 16±6.1 |  | 931±216 |  | 189±9.4 |  | 885 | 823 |  | 824 | 884 |  |
| Marginal | 22 | 7.2 - 37 | 15±5.8 | 0.83 | 870±228 | 0.22 | 192±10 | 0.17 | 12 | 10 | 0.80 | 9 | 13 | 0.49 |
| Failed | 22 | 1.7 - 21 | 14±4/7 | 0.29 | 925±210 | 0.89 | 190±9.5 | 0.66 | 11 | 11 | 0.87 | 12 | 10 | 0.56 |
| Contaminated | 17 | 4.3 - 23 | 14±5.0 | 0.27 | 916±252 | 0.79 | 193±10 | 0.12 | 13 | 6 | 0.15 | 10 | 9 | 0.70 |

1 Abbreviations: DBS, dried blood spot; Gest. Age, gestational age (days); BPD, bronchopulmonary dysplasia; P, P-value

2 Determined by Picogreen

3 Mean ± S.D.

4 P-values for DNA concentration, birth weight, and gestational age, were calculated using Student’s t-test with “Successful” as the reference; P-values for BPD and gender were calculated using chi-square analysis with “Successful” as the reference
