# Supplementary material for: High Quality Genome-Wide Genotyping from Archived Dried Blood Spots without DNA Amplification
Source: PLoS One. 2013 May 30;8(5):e64710. doi: 10.1371/journal.pone.0064710 (PMC3667813; doi:10.1371/journal.pone.0064710)
Supplement: Table S3 — Performance of 81 poorer performing gDNA samples on repeat DBS extraction and genotyping. (DOCX) [file pone.0064710.s004.docx]

| **Table S3**. Performance of 81 poorer performing gDNA samples on repeat DBS extraction and genotyping | | | | | | | |
| --- | --- | --- | --- | --- | --- | --- | --- |
|  |  | Sample performance on repeat extraction and genotyping | | | | | |
|  |  | Successful (CR >99.5%) | Successful (CR 99-99.5%) | Marginal | Failed | Contaminated | Total |
| Sample performance on original genotyping | Successful (CR 99-99.5%) | 25 | 5 | 0 | 0 | 0 | 30 |
|  | Marginal | 11 | 5 | 1 | 4 | 0 | 21 |
|  | Failed | 10 | 6 | 1 | 0 | 0 | 17 |
|  | Contaminated | 9 | 2 | 0 | 1 | 1 | 13 |
|  | Total | 55 | 18 | 2 | 5 | 1 | 81 |
