# Supplementary material for: High Quality Genome-Wide Genotyping from Archived Dried Blood Spots without DNA Amplification
Source: PLoS One. 2013 May 30;8(5):e64710. doi: 10.1371/journal.pone.0064710 (PMC3667813; doi:10.1371/journal.pone.0064710)
Supplement: Table S4 — Replication rate in DBS extraction and genotyping duplicates. (DOCX) [file pone.0064710.s005.docx]

| **Table S4**. Replication rate in DBS extraction and genotyping duplicates | | | | | | |
| --- | --- | --- | --- | --- | --- | --- |
|  | DBS | Consistent SNPs | Discrepant SNPs^1^ | Total SNPs^2^ | Replication rate^3^ | Mean replication rate (± S.D.) |
| Extraction duplicates | 1 | 2,396,439 | 100 | 2,396,539 | 0.999958 | 0.999950 ± 1.3E-5 |
|  | 2 | 2,397,510 | 90 | 2,397,600 | 0.999962 |  |
|  | 3 | 2,393,902 | 167 | 2,394,069 | 0.999930 |  |
|  | 4 | 2,398,009 | 106 | 2,398,115 | 0.999956 |  |
|  | 5 | 2,398,833 | 139 | 2,398,972 | 0.999942 |  |
| Genotyping duplicates | 6 | 2,389,096 | 148 | 2,389,244 | 0.999938 | 0.999949 ± 1.2E-5 |
|  | 7 | 2,401,199 | 101 | 2,401,300 | 0.999958 |  |
|  | 8 | 2,396,900 | 115 | 2,397,015 | 0.999952 |  |
|  | 9 | 2,394,519 | 125 | 2,394,644 | 0.999948 |  |
|  | 10 | 2,392,977 | 172 | 2,393,149 | 0.999928 |  |

1 Discrepancies across extraction and genotyping duplicates are not significantly different (P = 0.55, Student’s t-test)

2 SNPs successfully called in both the original and duplicate samples

3 SNP genotyping replication rate (GWAS discovery sample set)
