# Supplementary material for: High Quality Genome-Wide Genotyping from Archived Dried Blood Spots without DNA Amplification
Source: PLoS One. 2013 May 30;8(5):e64710. doi: 10.1371/journal.pone.0064710 (PMC3667813; doi:10.1371/journal.pone.0064710)
Supplement: Table S5 — Reagent costs for preparing gDNA and wgaDNA from DBS. (DOCX) [file pone.0064710.s006.docx]

| **Table S5.** Reagent costs for preparing gDNA and wgaDNA from DBS | | | | |
| --- | --- | --- | --- | --- |
|  | Reagent | Price | Cost per 96 extractions | |
| gDNA | 50 μg/μl Proteinase K | $110 (2 ml) | $5 |  |
|  | 2x Lysis Solution | $210 (0.5 L) | $3 |  |
|  | 96-well plates | $250 (50) | $10 |  |
|  | Capmat lids | $50 (50) | $2 |  |
|  | MPC Reagent | $560 (0.5 L) | $17 |  |
|  | GlycoBlue | $160 (1.5 ml) | $11 |  |
|  | Isopropanol | $30 (3 L) | $1 |  |
|  | Ethanol | $130 (2 L) | $2 |  |
|  | TE Buffer | $30 (70 ml) | $1 |  |
|  | 1-1000 μl tips | $50 (8 plates) | $20 |  |
|  | 1-200 μl tips | $50 (10 plates) | $5 |  |
|  | 0.1-10 μl tips | $50 (10 plates) | $1 |  |
|  | Sub-total $78 | | |  |
|  | Total cost (96 samples) = $78 (extraction) + $78 (iterations*) = $156 | | |  |
| wgaDNA | Amplification kit | $2200 (500 rxns) | $420 |  |
|  | 0.1-10 μl tips | $50 (10 plates) | $6 |  |
|  | Sub-total $426 | | |  |
|  | Total cost (96 samples) = $78 (extraction) + $426 (amplification) = $504 | | |  |

* For each plate of 96 extractions, there was approximately one plate of 96 iteration extractions
