# Supplementary material for: High Quality Genome-Wide Genotyping from Archived Dried Blood Spots without DNA Amplification
Source: PLoS One. 2013 May 30;8(5):e64710. doi: 10.1371/journal.pone.0064710 (PMC3667813; doi:10.1371/journal.pone.0064710)
Supplement: Text S1 — Dried Blood Spot (DBS) Genomic DNA Extraction Protocol. (DOCX) [file pone.0064710.s007.docx]

**SUPPLEMENTARY TEXT**

**Dried Blood Spot (DBS) Genomic DNA Extraction Protocol**

Preparation

1. Number and record each DBS in lab database or spreadsheet so DBS are easy to track.
2. Store DBS in -20^o^C freezer suitable for biohazardous materials.
3. Using a 2 mm diameter punch (mouse ear punch, Kent Scientific), collect five punches from a DBS into a well of a 96 well polypropylene 2.2 ml deepwell plate (Fisher Scientific). Wipe the inside of the punch with tissue paper and flame sterilize between each DBS to minimize cross-contamination.

DNA Extraction

1. Dilute 1 μl of 50 μg/μl Proteinase K (Epicentre or Roche) into 300 μl of Tissue and Cell Lysis Solution (Epicentre) for each sample. Apply 300 μl of this mix to each well. Cover with Capmat lid (Fisher Scientific), vortex quickly, and centrifuge the 96 well plate for 30 s or less to spin samples down. For all centrifugation steps, we used an Avanti J-25I super speed centrifuge (Beckman Coulter) and a two-plate swinging-bucket rotor (Beckman Coulter model JS-5.9), which can accommodate up to four 2.2 ml deepwell 96 well polypropylene plates, though only two plates were typically used at a time.
2. Incubate 1 hour at 65°C, shaking plate gently side-to-side every 10 min until punches look white and solution looks red. Centrifuge 30 s or less to spin samples down.
3. Remove lid and add 160 μl of MPC Protein Precipitation Reagent (Epicentre) to each well. Replace lid and vortex vigorously for 10 s. Do not centrifuge. Chill on ice for 30 min or more.
4. Pellet debris by centrifugation for 60 min at ~6,500 x g.
5. Remove lid and pipette supernatants into a clean plate. (Use of a multichannel pipette is recommended, but requires practice.) Discard the pellets.
6. Dilute 1 μl of GlycoBlue (Ambion) into 400 μl of isopropanol for each sample. (GlycoBlue is a colored glycogen derivative that aids in visualization of the DNA pellet.) Add 400 μl of the mix to each well of recovered supernatant. Add a new Capmat lid and invert the plate 7-8 times to mix.
7. Incubate at –20°C for one hour.
8. Pellet the DNA precipitate by centrifugation for 60 min at ~6,500 x g.
9. Remove lid and carefully pipette off the isopropanol, taking care not to dislodge the DNA pellet. Wash pellet with 400 μl of 70% ethanol.
10. Carefully pipette ethanol off of pellet.
11. Allow pellet to air dry 1 hour. Pellet may still be slightly wet. Resuspend pellet in 20 μl of 10 mM Tris-HCl (pH 8) / 1 mM EDTA (TE buffer). Allow pellets to dissolve in buffer for 15 min or more, then pipette up and down to fully dissolve. Transfer solution to final plate or tube. (Store samples at -20^o^C if they will not be used immediately.)

DNA Quantification and Extraction Iterations

1. Follow manufacturer's instructions for Qubit (Invitrogen) DNA quantification using 1 μl of each sample.
2. Determine how many more extractions (iterations) of each DBS are necessary using the following chart:

| gDNA concentration (ng/μl) | Number of additional extraction wells |
| --- | --- |
| 9 or more | 0 |
| 5 – 8.9 | 1 |
| 3.3 – 4.9 | 2 |
| 2.5 – 3.2 | 3 |
| 2 – 2.4 | 4 |
| 1.6 – 1.9 | 5 |

1. Add five 2 mm punches of each DBS to each extraction well, using the number of wells calculated in step 2 above. (If there was not enough DBS for 5 punches in each well, we used 4 punches or fewer in each well, but did not decrease the number of wells.)
2. Repeat “DNA Extraction” steps 1 through 10.
3. Use original extraction sample to resuspend the new pellets of gDNA obtained from the same DBS. This increases the concentration of gDNA without changing the sample volume.
4. Quantify DNA again as in step 1.
